# New evidence of Yangtze delta recession after closing of the Three Gorges Dam

**DOI:** 10.1038/srep41735

**Published:** 2017-02-01

**Authors:** X. X. Luo, S. L. Yang, R. S. Wang, C. Y. Zhang, P. Li

**Affiliations:** 1 Institute of Estuarine and Coastal Research, School of Marine Sciences, Sun Yat-sen University, Guangzhou 510275, China; 2 State Key Laboratory of Estuarine and Coastal Research, East China Normal University, Shanghai 200062, China; 3 State-province Joint Engineering Laboratory of Estuarine Hydraulic Technology, Guangzhou 510275, China; 4 Guangdong Provincial Key Laboratory of Marine Resources and Coastal Engineering, Guangzhou 510275, China; 5 The Second Institute of Oceanography, State Oceanic Administration, Hangzhou 310012, China; 6 Survey Bureau of Hydrology and Water Resources of Changjiang Estuary, Shanghai 200136, China

## Abstract

Many deltas are likely undergoing net erosion because of rapid decreases in riverine sediment supply and rising global sea levels. However, detecting erosion in subaqueous deltas is usually difficult because of the lack of bathymetric data. In this study, by comparing bathymetric data between 1981 and 2012 and surficial sediment grain sizes from the Yangtze subaqueous delta front over the last three decades, we found severe erosion and significant sediment coarsening in recent years since the construction of Three Gorges Dam (TGD), the largest dam in the world. We attributed these morphological and sedimentary variations mainly to the human-induced drastic decline of river sediment discharge. Combined with previous studies based on bathymetric data from different areas of the same delta, we theorize that the Yangtze subaqueous delta is experiencing overall (net) erosion, although local accumulation was also noted. We expect that the Yangtze sediment discharge will further decrease in the near future because of construction of new dams and delta recession will continue to occur.

Since the mid-Holocene, delta progradation has played an important role in the history of human society. However, deltas are currently threatened by erosion and subsidence because of sediment starvation and sea-level rise^1^. In many rivers, including the Nile, Mississippi, Yellow, and Indus Rivers, the sediment discharge to the sea drastically decreased in recent decades because of dam construction, water diversion and/or soil conservation^2^,^3^,^4^,^5^. Because longshore currents can generally transport large amounts of sediment away from the delta area^6^,^7^, net delta erosion is expected to occur when the riverine sediment discharge becomes lower than the rate at which sediment is removed by longshore currents (RSLC). Delta recession resulting from net erosion and subsidence not only decreases the land area but it can also damage benthic habitats, destroy buried oil and gas pipes and optic cables, and devastate coastal engineering facilities, such as seawalls and docks. Accordingly, detecting or forecasting delta recession is very important for ensuring delta sustainability. Unfortunately, because of the difficulties associated with systematic surveys, evidence of delta recession is usually lacking. This is particularly true for large subaqueous deltas.

The Yangtze is among the world’s five largest rivers in terms of length, water discharge and sediment load. A total of 450 million people live within this river’s watershed^8^. Since the 1950s, more than 50,000 dams have been built within the Yangtze watershed for power generation, water extraction and flood control, and many others are under construction^9^,^10^. Among them, the Three Gorges Dam (TGD), which was put into use in 2003, ranks as the world’s largest dam and substantial efforts have been expended to investigate its environmental impacts^11^,^12^,^13^,^14^,^15^,^16^. In addition to dam construction, soil conservation since the 1990s has also reduced the Yangtze’s sediment discharge^8^,^13^,^17^. In recent years (2003–2015), the Yangtze’s sediment discharge has decreased to below 140 Mt/yr, which is only 27% of its pre-decline level in the 1960s (510 Mt/yr)^18^ and is also lower than its original level in 2000 years before present (BP) (240 Mt/yr), when the sediment yield began to increase because of human activities^19^. Controversy exists regarding the recent morphological trends of the Yangtze subaqueous delta. Yang *et al*.^9^ found that the progradation rate in the Yangtze subaqueous delta decreased with riverine sediment discharge during the second half of the last century and that rapid recession has occurred since the TGD began to operate. Conversely, Dai *et al*.^20^ reported a rebound of high accumulation after the construction of the TGD, which is inconsistent with the decreased sediment supply. Other studies have shown slight accretion^21^ or moderate erosion^22^ in recent years. The disagreement among these studies is attributable to the fact that these studies were conducted on different areas or during various periods. Most importantly, bathymetric data are usually lacking for the outer margin of the Yangtze subaqueous delta^9^,^20^,^21^,^22^.

To ascertain the trends of progradation or recession in the Yangtze subaqueous delta front (Fig. 1), in this study, we determined recent accretion/erosion trends using bathymetric data collected in 1981, 1997 and 2012 from a previously unstudied area in the Yangtze subaqueous delta front and examined the temporal change in the grain size of surficial sediments in the transitional zone between the subaqueous delta and the Pleistocene sand seabed over the last 30 years. Our objectives were to determine: 1) whether net erosion has occurred in the new study area and 2) whether the border between the subaqueous delta mud and the Pleistocene relic sand has retreated landward in recent years.

## Results

### Temporal changes in influencing factors

Interannual fluctuations in the factors that influence the aggradation/degradation of the Yangtze subaqueous delta between 1981 and 2012 were significant (Fig. 2). However, fluvial sediment discharge showed a strong decreasing trend, whereas wind speeds, wave height, and sea level at the delta front showed only slight increasing trends over this period. In contrast, although the tidal range showed no trend, it exhibits a well-defined cyclicity with a period of approximately 19 years (Fig. 2, Table 1). No trend in wind direction was found.

### From aggradation to degradation in the North Branch delta front

**Figure 1:**
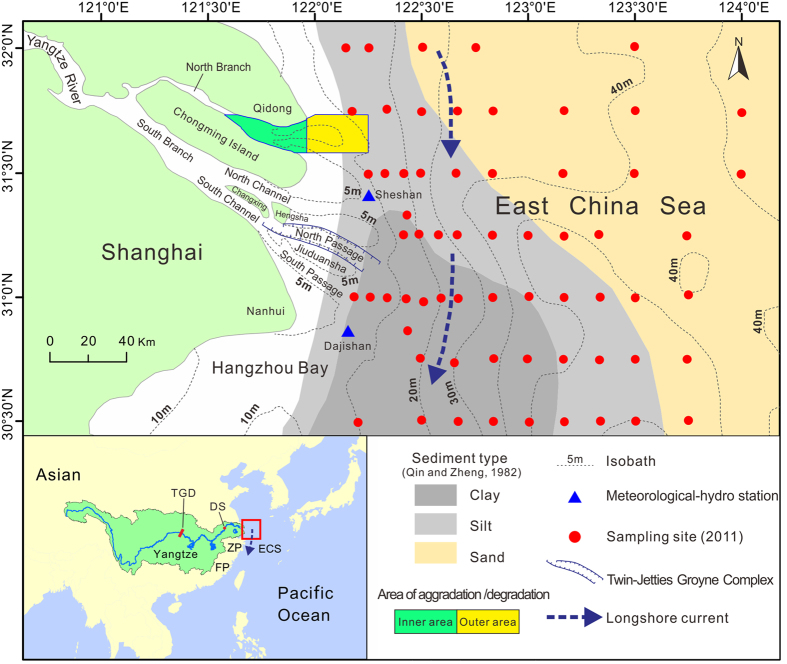
Map of the study area showing the scope of the Yangtze subaqueous delta (excluding the sand seabed) and sampling sites. ECS: East China Sea; ZP: Zhejiang Province; FP: Fujiang Province; TGD: Three Gorges Dam, DS: Datong Station. The maps were created using ArcGIS 10.1 (http://www.esri.com/software/arcgis) and CorelDRAW Graphics Suite X6 (http://www.coreldraw.com/en/product/graphic-design-software/).

**Figure 2:**
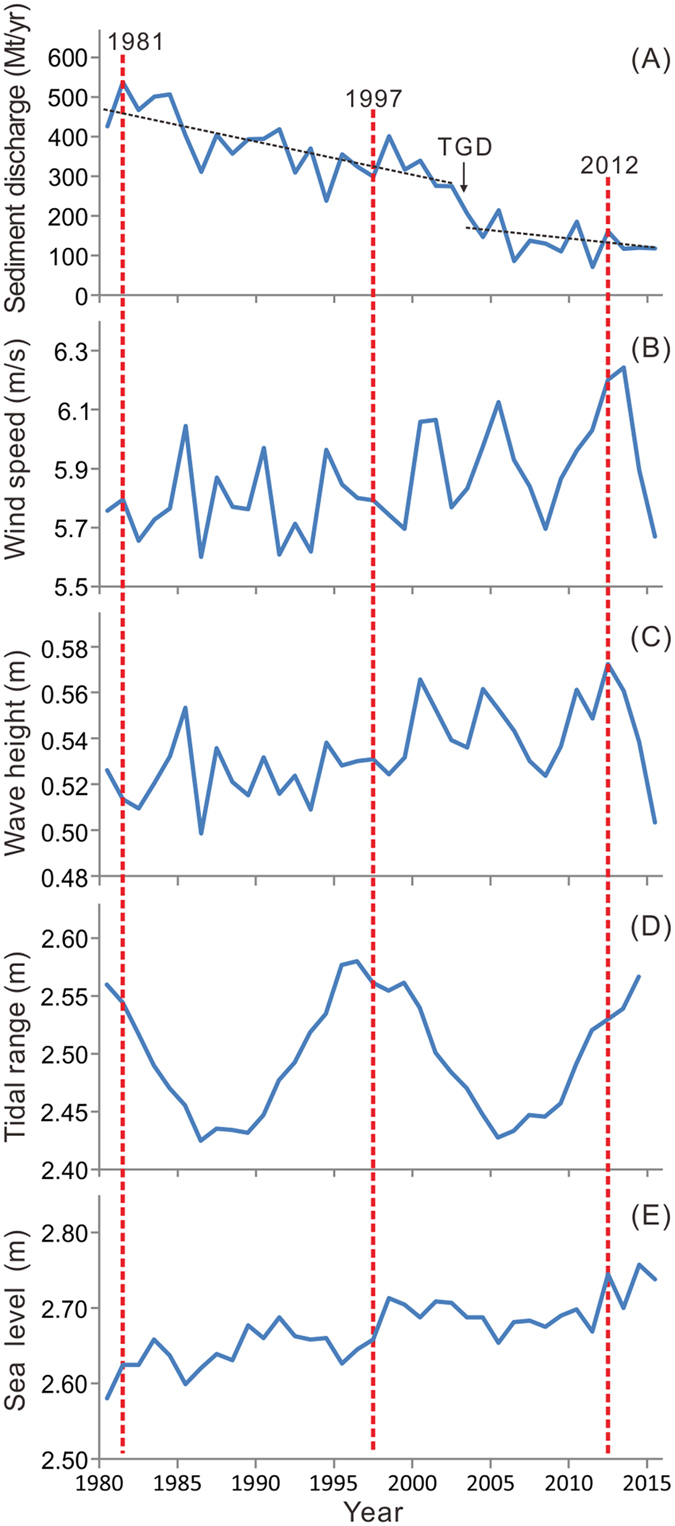
Temporal changes in sediment discharge, wind speed, wave height, tidal range and sea level (1980–2015). (**A**) Annual sediment discharges from the Yangtze River to the sea (Datong Station) showing an overall drastic decreasing trend between 1980 and 2015 and a abrupt decline in 2003 when the Three Gorges Dam (TGD) was put into operation. (**B**) Annually averaged wind speeds in the Yangtze’s subaqueous delta front (Sheshan Station) showing a slight increasing trend. (**C**) Annually averaged significant wave heights (Sheshan Station) showing a slight increasing trend. (**D**) Annually averaged tidal ranges (Sheshan Station) showing a periodicity of ca. 19 years but no increasing/decreasing trend. (**E**) Annually averaged sea levels (above the Astronomic Lowest Tide) in the Yangtze subaqueous delta front (Dajishan Stations) showing an increasing trend.

**Figure 3:**
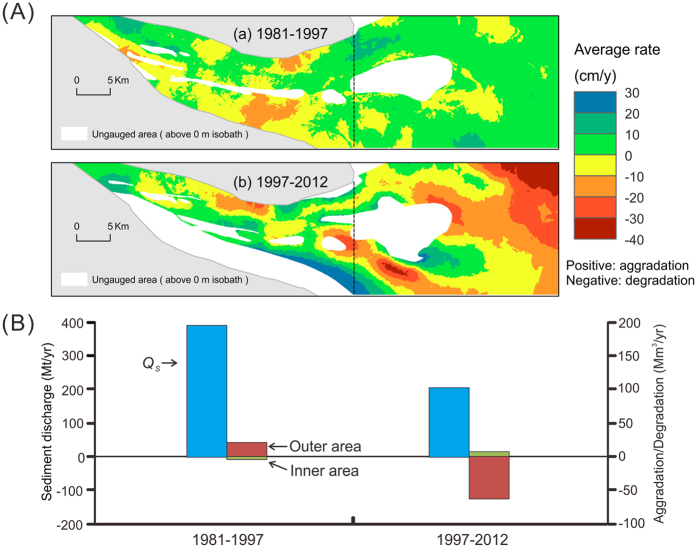
Changes in aggradation/degradation rates in the lower North Branch and adjacent subaqueous delta front in comparison with the Yangtze sediment discharge. (**A**) Spatial distribution of net aggradation/degradation rate over periods 1981–1997 and 1997–2012. (**B**) Period averaged sediment discharges from the Yangtze River (Datong Station) and aggradation/degradation rates in the lower North Branch and adjacent subaqueous delta front, showing similar decline trends. The maps were created using ArcGIS 10.1 (www.esri.com/software/arcgis) and CorelDRAW Graphics Suite X6 (http://www.coreldraw.com/en/product/graphic-design-software/).

The North Branch is one of the four outlets of the Yangtze River. The study area around the mouth of the North Branch constitutes ca. 1100 km^2^, including an inner portion (450 km^2^) and an outer portion (650 km^2^) (Fig. 1). During 1981–1997, although the annual sediment discharge of the Yangtze River showed a decreasing trend, the period-averaged sediment discharge was relatively high (390 Mt/yr), and the mouth area of the North Branch experienced overall aggradation, with an average accretion rate of 1.6 ± 0.02 cm/yr. During 1997–2012, however, the period-averaged sediment discharge of the Yangtze River decreased to less than 200 Mt/yr, mainly due to the construction and the use of the TGD (Fig. 2A), and the mouth area experienced degradation, with an average degradation rate of −6.7 ± 0.06 cm/yr. Degradation in the late period was mainly found in the outer area, i.e., the delta front, where the mean degradation rate was −11 ± 0.1 cm/yr, and the maximum degradation rate reached −40 ± 0.3 cm/yr (−6 ± 0.1 m during the 15-year period) (Fig. 3A).

### Coarsening of surface sediments at the subaqueous delta margin

**Figure 4:**
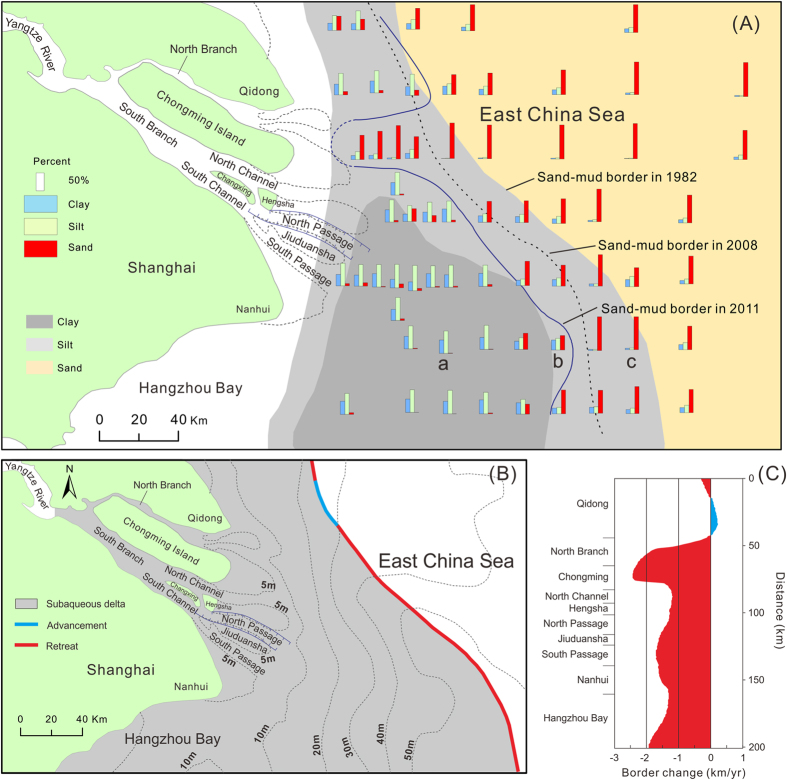
Changes in sand–mud border between the Yangtze’s subaqueous delta and the Pleistocene sand sheet in the East China Sea, which suggest rapid recession in the outer margin of the main delta. (**A**) Histograms of sediment composition and the sand–mud border (determined by interpolation) based on sampling from 2011 (post-TGD) and the sand–mud borders based on sampling from 2008 (also post-TGD)^45^ and before 1982 (pre-TGD)^46^. a, b and c in this figure show the sampling sites corresponding to the typical grain size frequency distributions shown in Fig. 5. (**B**) Map of net retreat and advancement sectors of the outer delta margin between 1982 and 2011. (**C**) Graph of sand–mud border change rate (error ± 0.1 km/yr) between 1982 and 2011. The maps were created using ArcGIS 10.1 (www.esri.com/software/arcgis) and CorelDRAW Graphics Suite X6 (http://www.coreldraw.com/en/product/graphic-design-software/).

**Figure 5:**
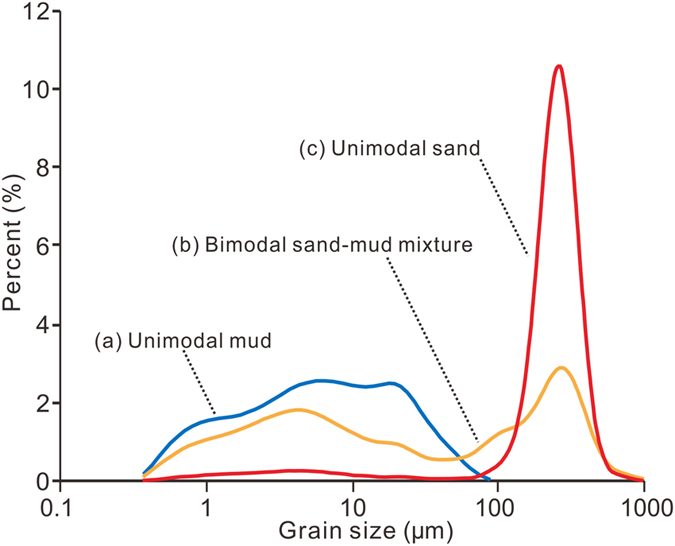
Typical grain size frequency distributions in the mud, sand and transitional zones based on sediment sampling in 2011. Curve (a) reflects pure mud composition in the Yangtze subaqueous delta; Curve (c) reflects pure sand composition in the Pleistocene sand sheet; and Curve (b) reflects mixture of the two sediment sources at the current sand–mud border. The sampling sites of a, b and c are shown in Fig. 4A.

The overall spatial pattern of surface sediment grain size in the study area has only slightly changed over the past three decades, and the sediments in the area close to the river mouth are muddy, whereas further offshore, in the East China Sea, the sediments are sandy (Fig. 4A). However, at the outer margin of the modern subaqueous delta that has developed in the area off the present-day river mouths, the sand–mud border has retreated rapidly landwards (Fig. 4A). The average retreat rate in this area was around 1.5 km/yr from 1982 to 2011, and a maximum retreat rate of approximately 2.3 km/yr was recorded in the area off the North Branch mouth. In comparison, little change was found in the sand–mud border north of the North Branch mouth, and the rapidly retreating section accounts for about 80% of the total length of the sand–mud border in the study area (Fig. 4B,C). The grain size distribution of the sediment sampled near the sand–mud border was bimodal, whereas the sediments sampled further away from the sand–mud border showed an unimodal distribution (Fig. 5).

## Discussion

The subaqueous delta off the Yangtze River mouth exceeds 10,000 km^2^ in area (Fig. 1)^23^. However, in previous studies, the aggradation/degradation rate of this delta was determined based on bathymetric data that covered only 10% to 20% of the total area. Specifically, the previous study areas were limited to the shallow areas (<20 m in water depth) off the three mouths of the South Branch^9^,^20^,^21^,^22^ (Fig. 1). Consequently, across the majority of the delta, the recent morphological trends remain unknown. In the current study, we found that the North Branch delta front has switched from aggradation to rapid degradation in recent years. As shown in the northeast of Fig. 3A, an aggradation rate of 10 ± 0.1 cm/yr between 1981 and 1997 was replaced by a degradation rate of −40 ± 0.3 cm/yr between 1997 and 2012. The recent landward retreat of the sand–mud border along the modern subaqueous delta margin (Fig. 4) also suggests delta recession. It can be reasonably supposed that the mud layer at the modern delta margin was thin. This hypothesis is supported by a cross-shore high-resolution seismic profile collected by Xu *et al*.^24^. The cross-shore profile was surveyed off the mouth of the North Passage after 2003. This seismic profile shows that the thickness of the overlying mud wedge decreases from 40 m at the nearshore starting point to zero at the offshore end point (50-m isobaths)^24^. It is likely that the thin mud layer was recently eroded, and the Pleistocene sand became exposed. The bimodal grain size distribution (Fig. 5) probably reflects the mixture of exposed sand and eroding mud at the recent sand–mud border. Notably, the strongest sediment coarsening was found at the delta front just outside the North Branch (Fig. 4), where severe erosion has occurred (Fig. 3). So, we found morphological and sedimentary evidence of recession in previously unstudied areas of the Yangtze delta.

The Yangtze delta has advanced southeastwards over the Holocene, and the modern subaqueous delta has formed mainly over the past 200 years. The coast north of the North Branch mouth formed earlier than the modern Yangtze delta^19^,^23^. Because of the influence of the monsoon and the geostrophic force, transport of sediment from the Yangtze River has occured mainly southwards on the continent shelf^6^,^23^. Accordingly, little sediment from the Yangtze River has been deposited in the coastal waters north of the North Branch over the recent centuries. This argument is supported by the change in location of the sand–mud border, which was much closer to the coastline in the section north of the North Branch mouth in 1982 than in the section off the current river mouths (Fig. 4A). It is reasonable to suppose that under a regime of sediment starvation and hydrodynamics increase less erosion would occur at locations characterised by low deposition. This explains why the rapid retreat of the sand–mud border has occurred at the modern subaqueous delta margin, but not in the section north of the North Branch. Although the retreat of the sand–mud border suggests delta margin recession, the rate of the sand–mud border retreat can be different from the rate of isobath recession, because the former derives from grain size evidence, whereas the latter is based on bathymetric data. The transformation from muddy to sandy seabed can be caused either by full erosion of the mud layers that covered the sand, or by selective erosion that exports preferentially fine-grained materials and forms a sand armouring at the surface.

Delta recession can be driven by a large number of processes, such as sediment starvation, sand mining, subsidence, and sea level rise, as well as an increase in current and wave energy^1^,^25^. As shown in Fig. 2A, sediment discharge from the Yangtze decreased by nearly 50% between 1981–1997 and 1997–2012. Meanwhile, wind speeds and wave heights increased by 2 to 4% (Fig. 2B,C), which suggests that sediment resuspension and residual sediment transport may have increased slightly. However, the contribution of increased winds and waves to coastal recession in this subaqueous delta over the past three decades is most probably minor in comparison with the impact of decreased sediment discharge. The 18.6 year tidal cycle can significantly contribute to regional coastal changes^26^. In this study, the annually mean tidal range fluctuated between 2.4 and 2.6 m because of the 18.6 year cyclicity (Fig. 2D). Thus, this 18.6 year tidal cycle may have probably played a role in the 5–10 year coastal evolutions. Nevertheless, the contribution of tidal change to delta recession over the past three decades has most likely negligible, considering the average tidal range in period 1997–2012 was equal to the average tidal range in period 1981–1997. Sea level has increased at a rate of about 3 mm/yr over the past three decades (Fig. 2E). As the tide gauge stations were constructed on rock substrates, this local sea level rise reflects the combination of isostatic depression and global sea level rise. In addition, sediment compaction resulted in an additional subsidence of about 3 mm/yr^27^ in the Yangtze delta. Together, these factors would result in coastal recession at a rate of around 6 m/yr, because the coastal gradient is approximately 1‰ (Fig. 1) in this area, unless it was offset by sediment deposition. This recession has important implications for intertidal wetlands. However, its contribution to the retreat of the sand–mud border is negligible, considering the latter was much higher in magnitude and the latter was deduced from grain size data rather than bathymetric data. The subsidence caused by sediment compaction would have increased degradation and reduced aggradation. For example, the accretion rate of 1.6 cm/yr over the period 1981–1997 and the degradation rate of −6.7 cm/yr over the period 1997–2012, calculated above for the mouth area of the North Branch, suggest a sediment accumulation rate of 1.9 cm/yr and a sediment erosion rate of −6.4 cm/yr, respectively. In the delta front off this channel mouth, the average degradation rate of −11 cm/yr over the latter period suggests an erosion rate of −10.7 cm/yr. This suggests that more than 95% of the degradation at this delta front can be attributed to erosion. Thus, sediment starvation is undoubtedly the main cause of the recession of this delta. Given that sediment discharge from the Yangtze has decreased abruptly since 2003 (Fig. 2A), when the TGD become operational^15^,^28^, the recession of the delta has occurred most probably during the post-TGD period.

It is possible that, after the TGD and numerous other water projects within the Yangtze watersheds were implemented^8^, erosion occurred somewhere in the immense Yangtze delta, whereas accumulation continued elsewhere in the delta. The most important cause of continued accumulation is large-scale accumulation-promoting projects conducted in the coasts at the mouth area of the Yangtze River^20^,^21^. The Twin-Jetties Groyne Complex, which was constructed to maintain the Deep Waterway in the North Passage, also attenuated hydrodynamics and increased accumulation in surrounding areas^21^,^29^. In addition, changes in the allocation of riverine water and sediment discharge among the bifurcated channels in the Yangtze Estuary may also have resulted in migration of the deposition center^20^.

One important issue is the overall erosion/accumulation trend, or net erosion/accumulation, in the entire subaqueous delta off the Yangtze River mouth since the closure of the TGD and in the future. Although this question cannot be directly answered based on morphological changes because of the lack of bathymetric data, a large-scale sediment budget approach for the Yangtze Delta and the inner continental shelf of the East China Sea should help examine the overall erosion/accumulation trend of the Yangtze subaqueous delta. Driven by the Eastern Asian Monsoon, a strong southward longshore current flows through the Yangtze subaqueous delta in winter^30^. More than 150 Mt of sediment can be delivered annually by this longshore current system from the Yangtze subaqueous delta^6^. Investigations of the deposition rate in the Holocene mud wedge in the inner continental shelf along the coasts of the Zhejiang Province (ZP) and Fujiang Province (FP) (Fig. 1) support this conclusion^31^,^32^. A recent study revealed that the longshore currents can transport sediment away from the Yangtze subaqueous delta at a rate of 270 Mt/yr^33^.

Since the full operation of the TGD began in 2010, the sediment discharge from the Yangtze River has decreased to 130 Mt/yr. Considering the sediment retained by the accumulation-promoting structures in the Yangtze Estuary^21^,^22^ and the sediment naturally deposited in the nearly abandoned North Branch^34^ and in salt marshes^9^, the actual sediment discharge to the subaqueous delta off the river mouth has most likely been below 100 Mt/yr, which is significantly lower than the rate at which sediment is transported away from the Yangtze subaqueous delta by the longshore currents. Thus, overall erosion has probably occurred in the Yangtze subaqueous delta. Any instance of accumulation exceeding the sediment discharge from the river implies erosion elsewhere. For example, Dai *et al*.^20^ reported an accumulation rate of nearly 500 × 10^6^ m^3^/yr (or ca. 600 Mt/yr) in the Yangtze River mouth and nearby areas in 2002–2004 (Fig. 4 in that study). In fact, the sediment supply from the Yangtze River in 2002–2004 was only 210 Mt/yr. This difference suggests an erosion rate of ca. 400 Mt/yr in unknown nearby areas, not including the large amount of sediment expected to be transported away from the Yangtze subaqueous delta by the longshore currents.

In the near future, the Yangtze sediment discharge to the sea will likely decrease to below 100 Mt/yr because of the construction of new dams and the South to North Water Diversion^28^. As a result of engineering-generated retention and natural sediment deposition in the estuary, the rate at which sediment reaches the subaqueous delta front will probably be less than half of the rate at which sediment is transported away from the delta area by the longshore currents. Thus, continued and stronger erosion in the Yangtze subaqueous delta front is expected.

## Study Area

The Yangtze River originates on the Qinghai-Tibet Plateau at 5100 m above sea level and flows 6300 km eastward to the East China Sea. Since ca. 2000 years BP, deforestation for cultivation in the Yangtze Basin more than doubled the sediment discharge to the sea until the mid-20^th^ century^19^,^35^ and accelerated delta progradation^36^,^37^. However, this trend has been reversed since the 1960s when the Danjiangkou reservoir, then the largest and now the second largest reservoir after the TGD, was constructed on the Hanjiang River, a major source of sediment in the Yangtze River^38^. Because of the continuous construction of dams, the cumulative storage capacity of reservoirs within the Yangtze basin has increased by more than 5 times since the 1960s. Furthermore, since the 1990s, basin-scale soil conservation has been implemented within the Yangtze watershed. Altogether, these human activities have caused most of the sediment that would have been delivered into the sea to be retained^8^. As a result, the present sediment discharge of the Yangtze River (140 Mt/yr in 2003–2015) is far below its pre-human level prior to 2000 years BP (240 Mt/yr)^19^, which has implications for the delta morphology^9^.

The current Yangtze Estuary includes three bifurcations and four outlets (Fig. 1). The first bifurcation resulted from the formation of Chongming Island, whose embryo was first found 1400 years ago^37^. Prior to the 18^th^ century, the North Branch was the main outlet of the Yangtze water and its sediment discharge. In the 18^th^ century, however, the major river flow shifted to the South Branch. Since that time, the North Branch has become narrower and silted up. The volume of the North Branch decreased by 0.64 km^3^ between 1958 and 2010^33^, which suggests a mean accumulation rate of ca.16 Mt/yr, taking into account the dry bulk density of 1.3 g/cm^3^ for the Yangtze sediment^38^. It can be roughly estimated that more than 1 km^3^ (or 1300 Mt) of sediment has been trapped in the North Branch since its silting up that otherwise would have reached the subaqueous delta front. Thus, currently, less than 5% of the river discharge flows across the North Branch^23^,^34^. The South Branch has been split into the North and South Channels because of the formation of Changxing and Hengsha islands in the 17^th^ century. Subsequently, in the 1950s, the South Channel was divided into the North and South Passages because of the emergence of the Jiuduansha Shoal. In the high deposition area between 10-m and 30-m isobaths off the mouths of the South Branch, the accumulation rate was 3–5 cm/yr during the several decades prior to the 1980s^39^,^40^,^41^,^42^. Since the 1980s, however, accumulation has significantly slowed down as a result of the dramatic decrease in the sediment supply from the Yangtze River^41^.

In this subaqueous delta, sediment in the upper several metres is mud-dominated with a high water content and a low critical shear stress for erosion^43^. During spring tides, the current velocity can exceed 2.0 m/s^44^. The bed shear stress usually exceeds the critical shear stress for erosion, except during tidally slack waters and calm weather, suggesting frequent sediment resuspension^45^. The monsoon-driven winds are southeasterly in summer and northerly in winter, and the wind speed is greater in winter than in summer. In winter, a strong southward longshore current develops under the influence of northerly monsoon winds. Under typical northerly winds in winter (7–8 m/s speed), the residual longshore current velocity of the water column can exceed 40 cm/s^33^.

## Methods

### Datasets

Sediment discharge data from Datong Station were collected by the Changjiang (Yangtze River) Water Resource Committee (CWRC, http://www.cjw.gov.cn/zwzc/bmgb/nsgb). The suspended sediment load was used to represent the total sediment discharge because the Yangtze River bed load accounts for less than 1% of the total sediment load^41^. Data regarding wind direction and speed, as well as wave height, for Sheshan Station were obtained from the European Centre for Medium-Range Weather Forecasts (http://www.ecmwf.int/). Data related to the water level used to calculate the annual average tidal range and sea level at Sheshan and Dajishan stations were provided by the Ocean Bureau of China (http://www.soa.gov.cn/zwgk/hygb/). Bathymetric maps were obtained from the Maritime Survey Bureau of Shanghai, Ministry of Communications of China. The bathymetric data were collected in 1981, 1997, and 2012 using an echo sounder with a precision of 0.1 m; a global positioning system (GPS) device with a horizontal error of ±1 m was used for navigation. The bathymetric tidal corrections used tidal levels recorded at gauging stations within the area of bathymetric maps. The scales of the bathymetric maps for 1981, 1997, and 2012 were 1:120,000, 1:250,000, and 1:150,000, respectively, and the number of data points on the maps was 1232, 582 and 1005, respectively. The maps were processed using the ArcGIS software developed by Environmental Systems Research Institute (ESRI, USA). Each set of depth soundings was interpolated onto a grid composed of cells of 30 × 30 m using the Kriging interpolation technique, which produced more than 1,000,000 time series of bathymetric data. At each grid point, deduction of the later depth from the earlier depth provided the thickness of accretion (positive) or degradation (negative). Then, digitized maps of this depth change were used to calculate the vertical accretion/degradation rates and for delineating accretion/degradation areas^9^.

### Calculation of aggradation/degradation

Each set of bathymetric data was interpolated to a grid with 50 × 50-m cells using a kriging interpolation technique. Digitized maps were used to calculate the vertical accretion/erosion rates and to delineate the accretion/erosion areas. The total volumes of accretion and erosion and the difference between them were then calculated. The rate of annual accretion or erosion was then calculated by dividing the net volume by the area and time (years). The kriging interpolation technique is widely used in Geographic Information System (GIS) analyses. The error associated with the sediment volume based on bathymetry and the kriging interpolation technique is associated with the difference in depth between the neighbouring bathymetric data points and the complexity of the seabed morphology. As the difference in the depth between the data points and the complexity of the seabed morphology increase, the error associated with the calculated sediment volume also increases. In the current research, numerous data points are included in each bathymetric map (Fig. S1), and the seabed of the subaqueous delta is smooth, with gradients typically <1‰ (Fig. 1). Therefore, the errors associated with sediment volume estimation using kriging interpolation are assumed to be very low (<1%, as estimated).

### Sediment sampling and analysis

To examine the recent changes in the spatial patterns of sediment in and around the outer Yangtze subaqueous delta, we obtained surficial sediment samples (upper 30 cm) using a box sampler in April 2008 and July 2011. In the laboratory, the grain-size distributions of the sediment were determined using a laser particle size analyser (LS-100Q, Beckman Coulter Corporation, USA), which subdivides each sample into 117 size fractions between 0.0002 mm and 2 mm. Prior to grain-size analysis, the organic matter and carbonate were removed from the sediment samples using H_2_O_2_ and HCl. The aggregates were then dispersed by the addition of (NaPO_3_)_6_ and subsequent ultrasonic treatment. The sand–mud border in the East China Sea was determined by interpolation. The sand percentage was >50% on the seaward side of the sand–mud border, and the mud (silt and clay) percentage was >50% on the landward side of this border.

## Additional Information

**How to cite this article:** Luo, X. X. *et al*. New evidence of Yangtze delta recession after closing of the Three Gorges Dam. *Sci. Rep.* **7**, 41735; doi: 10.1038/srep41735 (2017).

**Publisher's note:** Springer Nature remains neutral with regard to jurisdictional claims in published maps and institutional affiliations.

## Supplementary Material

Supplementary Figures

## Figures and Tables

**Table 1:** Annual and period-averaged values of factors that influence aggradation/degradation of the Yangtze subaqueous delta.

	1981	1997	2012	1981–1997	1997–2012
Sediment discharge (Mt/yr)	540	300	160	390	210
Wind speed (m/s)	5.8	5.8	6.2	5.8	5.9
Significant wave height (m)	0.51	0.53	0.57	0.52	0.54
Tidal range (m)	2.54	2.56	2.53	2.50	2.50
Sea level (m)	2.62	2.66	2.74	2.64	2.69
