# Supplementary material for: New evidence of Yangtze delta recession after closing of the Three Gorges Dam
Source: Sci Rep. 2017 Feb 1;7:41735. doi: 10.1038/srep41735 (PMC5286395; doi:10.1038/srep41735)
Supplement: Supplementary Figures [file srep41735-s1.pdf]

New evidence of Yangtze delta recession after closing of the Three Gorges Dam

X.X. Luo, S.L. Yang, R.S. Wang, C.Y. Zhang, P. Li

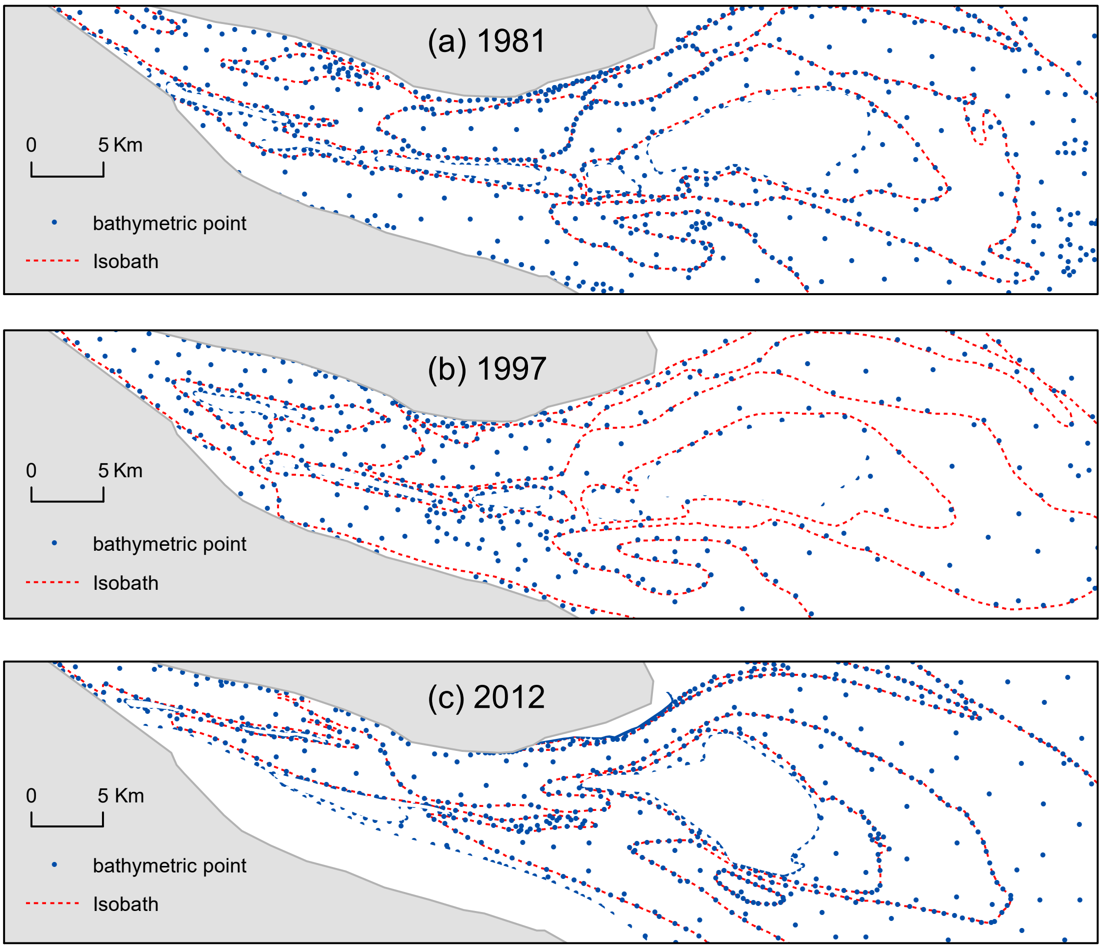

Figure S1 Data points on the bathymetric maps of the study area around the mouth of the North Branch. The maps were created using ArcGIS 10.1 (www.esri.com/software/arcgis).
